# Strengthening polio vaccine demand in Ghana: Understanding the factors influencing uptake of the vaccine and the effectiveness of different message frames

**DOI:** 10.1371/journal.pone.0279809

**Published:** 2023-02-10

**Authors:** Anna-Leena Lohiniva, Anastasia Nurzhynska, Abdul Mueed, Absar Ali, Khadeeja Ahmed, Paul Ayiku, Joshua Amo-Adjei, Yoshito Kawakatsu, Mrunal Shetye, Karen Greiner, Ross McIntosh

**Affiliations:** 1 Social and Behaviour Change Section, UNICEF Ghana Country Office, Accra, Ghana; 2 Cogent, Lahore, Pakistan; 3 Cogent, Geneva, Switzerland; 4 Cogent, Islamabad, Pakistan; 5 VIAMO, Accra, Ghana; 6 Department of Population and Health, University of Cape Coast, Cape Coast, Ghana; 7 Department of Data and Analytics, UNICEF HQ, New York, New York, United States of America; 8 Health Section, UNICEF Ghana Country Office, Accra, Ghana; 9 Social and Behaviour Change Section, UNICEF West and Central Africa, Dagar, Senegal; 10 UNICEF Polio, UNICEF HQ, New York, New York, United States of America; Texas A&M University, UNITED STATES

## Abstract

**Background:**

Ghana has experienced recent polio outbreaks. Behavioral insights can be used to understand behavior and create demand for the polio vaccine.

**Methods:**

This cross-sectional study is based on an interactive mobile phone survey that explored factors influencing the uptake of the polio vaccine among Ghanaian mothers with children younger than five years old. The survey also explores the mothers’ intention to vaccinate their children in the future as well as an experiment with short polio vaccine voice message nudges to identify the most effective message frames in encouraging vaccination. The study sample was drawn from volunteers from a mobile service platform. Linear probability model regressions with Ordinary Least Squares (OLS) estimates were used to analyze the data.

**Results:**

In total, data from 708 caregivers was assessed. Out of the sample, 35% (n = 250) had not vaccinated their children against polio, around 8% (n = 53) of respondents stated they did not plan to do so, while 28% expressed intent to do so during the next polio vaccination campaign. Higher vaccination of children against polio, i.e. better uptake of the polio vaccine, appeared to be associated with children’s caregivers knowing that polio causes paralysis (with a coefficient of 0.13 (95% CI: 0.02, 0.24), i.e. 13% more likely than not to have their child vaccinated). Higher vaccine uptake also appeared to be associated with the perception that the polio vaccine is safe (with a coefficient of 0.11 (95% CI: 0.01, 0.22), i.e. 11% more likely than not to have their child vaccinated). Another factor in increasing vaccine uptake is whether caregivers receive support from healthcare workers with a coefficient of 0.11 (95% CI: 0.02, 0.20), i.e. 11% more likely than not to have their child vaccinated. Crucially, difficulty accessing the polio vaccine appeared to be associate with a negative change in vaccine uptake (with a coefficient of -0.16 (95% CI: -0.23, -0.08), i.e. 16% less likely to have their child vaccinated). Satisfaction with the information provided by vaccinators was also associated with better vaccine uptake (with a coefficient of 0.12 (95% CI: 0.05, 0.20) i.e. 12% more likely than not to have their child vaccinated); and having seen or heard something negative about the polio vaccine with a coefficient of 0.10 (95% CI: 0.03, 0.17), i.e. 10% more likely than not to have their child vaccinated. The social norms message frame was statistically significant with a coefficient of 0.06 (95% CI: -0.004, 012).

**Conclusion:**

The findings from this study suggest that most women with children under the age of 5 appear to have vaccinated their children against polio. Many more caregivers express an intention to vaccinate their children, never having done so before. The behavior and the intention to vaccinate are both driven by a number of factors that must be addressed to create demand for the polio vaccine. Targeted message frames appeared to be statistically significant drivers of vaccine uptake. However, more research is required to understand how they impact vaccine behavior and future intention for vaccination.

## Introduction

### Polio situation

Poliomyelitis is a viral disease that can lead to paralysis and even death. It remains a public health concern worldwide although progress to eliminate the virus has been significant [1]. Since the global polio eradication efforts began in 1988, the incidence of polio cases has decreased by 99.99%. The WHO Africa Region was certified free of wild poliovirus (WPV) in August 2020. However, since 2017 cases of circulating vaccine-derived poliovirus 2 (cVDPV2) have been spreading worldwide, including in Africa. This has been explained by low immunization rates in communities [2,3]. Globally cVDPV2 has caused more cases of poliomyelitis than wild poliovirus every year since 2017, with 1057 cases reported in 2020 [4].

### Polio and polio vaccination in Ghana

Mass polio vaccination campaigns have been implemented in Ghana since 2000 until the Regional Polio Certification Committee declared Ghana a polio-free country in 2015. Since then, polio vaccination has been offered to mothers in Ghana as part of the routine immunization package [5]. Despite the fact that Ghana has had relatively high coverage of the polio vaccine during the past few years (93%) [2,3], there were two major polio outbreaks in 2003 and 2008 that have been explained by gaps in immunization coverage [6,7]. In 2020, health authorities reported once again 12 cases of cVDPV2 in 12 out of 16 regions, highlighting the importance of continued and intensified efforts to fight the virus. Poliovirus importation from neighboring countries with polio outbreaks such as Nigeria prior to global eradication also remains a threat in Ghana without long-term surveillance in place [6]. Accordingly, mass vaccination campaigns were launched in Ghana again in 2020 to address cVDPV2 outbreaks. The first round of the house-to-house campaign, during which vaccinators make house visits to vaccinate people, was conducted successfully in March 2020. However, global COVID-19 social distancing measures halted additional rounds of the campaigns.

### Introduction to novel nOPV2 vaccine

In 2022 Ghana was certified to use the novel nOPV2 vaccine and the first nOPV2 vaccine campaigns started in August 2022. This new vaccine provides intestinal immunity and is less likely to revert into a form that can cause paralysis than the oral polio vaccine that has previously used to fight the wild poliovirus [8].

The introduction of new vaccines can be challenging as the public may raise questions and express concerns, which can potentially lead to vaccine hesitancy and even refusal to take the vaccine. Vaccine hesitancy (being unsure about getting a vaccine) usually accounts for a more substantial share of the population who will not be vaccinated than those who are vaccine resistant (object to vaccines). Those who are vaccine-hesitant can often be influenced to take the vaccine as they are still thinking about it whereas those who refuse them entirely have made up their mind and accordingly are more resistant to vaccines. Therefore, vaccine campaigns often focus on those who are hesitant instead of trying to convince those who have decided not to take the vaccine [9].

### Factors influencing the uptake of polio vaccines

There is little knowledge about factors influencing the uptake of the polio vaccine or the impacts of previous campaign strategies and messages in Ghana. However, a number of socioeconomic and demographic factors have been identified that influence child immunization, highlighting the linkages between inequalities in health and vaccine coverage that include rural residence, low maternal education, unemployment [10,11], and difficult or no access to prenatal care [12]. Studies about childhood immunizations also highlight regional differences in the uptake of vaccines and suggest that the determinants of immunization uptake are linked to the lack of understanding of the benefits of immunization and parents’ fear of vaccines causing illnesses [10]. Being by religion a traditionalist or without religion has also been identified with lower odds of receiving complete vaccination, compared to children whose mothers were Christians [13]. However, immunization-related knowledge has been found to be relatively high among mothers indicating that lack of knowledge is unlikely to be the main reason for vaccine hesitancy or refusal [14].

### Behavioral insights

By understanding factors that influence the uptake of the vaccine, health authorities can develop appropriate strategies to encourage those who are hesitant to take the vaccine.

Behavioral science insights can be used to explore behavioral factors [15]. The Behavioral Drivers Model (BDM) was used as a conceptual framework for the study. The model categorizes factors influencing behavior into individual factors, social factors, and environmental factors, which in turn are divided into a number of dimensions. Based on the behavioral analysis, the framework provides suggestions for evidence-based interventions to modify the behaviors [16]. Theory-based interventions are known to be effective [17–19].

Behavioral sciences argue that people are not rational decision-makers but are influenced by various subconscious biases, which are there to help people make sense of the vast information flow. These cognitive biases can be used to design interventions to modify behaviors. They are called nudges, which aim to adapt the choice architecture for people by changing how choices are presented to them. The objective of nudging is to help people make better decisions for themselves without restricting their freedom of choice [20]. Nudging has been effectively applied in various health interventions such as in influenza vaccine promotion [21–23].

Different information frames can be used to nudge. Framing refers to the process by which people develop a particular conceptualization of an issue or reorient their thinking about an issue. A frame in communication refers to words, images, phrases, and a presentation style of information [24]. The literature highlights that different frames can influence differently the same information and the processing of that information including the intention to take an action. Frames are context-specific highlighting the importance of identifying the frame that works best with the intended behavior in a particular socio-cultural setting [24].

Communication about any vaccine is also transmitted through frames that are used in the messages [25]. For example, during the COVID-19 pandemic the media frequently used safety and efficacy frames [26,27] that are topics identified in a number of studies as some of the most common reason for vaccine hesitancy [28–30]. The use of social norms to change behavior has been long established [31,32]. Social norms refers to rules that members of a group recognize and that affect their decisions and behavior. Descriptive social norms are based on a principle that most people want to bring their behavior in line with what they perceive to be the behavior of others, whereas injunctive social norms tell people what is socially acceptable [31,33]. Social norms can also have a negative impact on behavior when the perception that everyone is not doing decreases the intention to take an action [33]. Various experiments have been conducted to test the impact of social norms on behavior change [34,35]. For example, social norms have been identified as the strongest predictors of the intent to be vaccinated against human papillomavirus (HPV) [36,37], as well as a factor that influences parental decisions to vaccinate children [38]. Healthcare workers are repeatedly identified as trusted sources of information which makes them ideal messengers of vaccine-related information both globally and in Ghana [39–41], whereas globally fact-framed messages have been found to be less effective [42].

Many message framing experiments to date have focused on loss gain frames by looking at the relative persuasiveness of the frames with mixed and sometimes even small effects [43–45]. Only a few vaccination related message framing studies have been conducted in Africa including a study in Nigeria that demonstrated immunization-related loss framing backfired [46]. Another study from Nigeria demonstrated that using a social norm-related messaging frame in the context of good parenting increased the intention of parents to immunize their children [47]. In Sierra Leone, a social signaling approach that framed messages based on the prevailing social norms of the society was effective: parents who fail to vaccinate their children are negligent [48]. A recent study in Ghana demonstrated that mobile phone-based messaging campaigns and incentives for healthcare workers and caregivers can increase the timely uptake of routine vaccinations [49]. To develop an effective vaccine demand creation plan there is a need to understand how different types of frames may influence the vaccination intentions.

### The aim of the study

UNICEF together with its partners Ghana Health Services (GHS); Cogent, a data analysis consultancy firm; and VIAMO, a global social enterprise specializing in mobile engagement and information and communication technology for development, initiated mobile phone-based surveys to explore determinants that influence polio vaccine uptake and the efficacy of nudges with different message frames in Ghana among mothers with children under five years old. Mothers were selected as a target audience as they have been identified as one of the main decision-makers in childhood immunization [50]. Mobile phone-based surveys, which are still a rather novel data collection method, were applied in the study in order to reach people from remote locations in Ghana. Interest towards mobile phone-based surveys is growing globally. They have been used in particular when collecting data from hard-to-reach populations such as homeless people [51]. Mobile phone-based nudges were used to encourage timely neonatal vaccination services in Ghana with some promising results [49]. However, to our knowledge this study is the first mobile phone-based survey in Ghana using Random Digit Dialing (RDD) to recruit study participants. The findings can be used to develop communication strategies, approaches and messages to create demand for the polio vaccine.

## Materials and methods

### Study design

The study consisted of two parts embedded in one mobile phone-based survey based on an interactive voice response (IVR) system, an automated system that delivers pre-recorded voice messages to people over mobile phones in their preferred language. The IVR system allows for two-way communication by sending voice messages to users who can send messages back to the IVR system by pressing specific numeric keys on their phone’s keypad. The first part of the study was a survey that explored factors influencing the uptake of the polio vaccine among mothers with children under five years old, and the factors influencing the intention of mothers who have not vaccinated their children to vaccinate their children against polio. The second part of the survey included an experiment to test which of several behaviorally informed message frames had the greatest effect on vaccine acceptance.

Part one of the survey measured predictors for behavior. These predictors measured various drivers of vaccination behavior and intentions, wherein respondents rated their perceptions on each of these drivers. The Behavioral Drivers Model (BDM) was used as a conceptual framework for the predictor development. The aim was to select predictors from all BDM categories: psychological, sociological, and environmental [16]. The selection was based on expert consensus. An earlier UNICEF Knowledge Attitude and Practice survey (KAP) was used to aid the development of the survey questions. See Table 1 for the BDM categories, selected BDM dimensions, and survey questions. Please see S1 Table for the full survey questionnaire tool.

**Table 1:** Behavioral Drivers Model (BDM) categories, dimensions, and related questions.

BDM category	BDM dimensions (predictors of behavior)	Questions
Psychological factors	Knowledge	Do you think polio causes paralysis? Do you think polio vaccines prevent polio? Have you seen or heard anything negative about polio vaccines? Based on your previous experience do you feel the vaccinators provide you with enough information about polio vaccination?
	Beliefs	Do you think the polio vaccine is safe? Do you think getting a polio vaccine will be important for the health of your child under five years old?
Sociological factors	Descriptive Norms	Do traditional/religious leaders in your community support children receiving polio vaccines? Do you think healthcare workers in your local health center support caregivers vaccinating their children against polio?
	Decision-making power	If it was time for your child to get the polio vaccination, would you need permission from your family members to vaccinate your child?
	Social support	Do you have household members who do not support children receiving the polio vaccine?
Environmental questions	Access and quality of services	Do you think that vaccinators provide enough information about the polio vaccine?
	Trust in service providers	How much do you trust the health care providers who would give your child a polio vaccine?
		Have you seen or heard anything negative about polio vaccines?

Part two of the survey focused on measuring the effectiveness of four short audio message nudges against a fact-based control message, each with different content, on two dependent variables: willingness to give the polio vaccine to children and willingness to recommend the polio vaccine to others. The nudges represented different conditions, namely social norms, perception of safety, communicating adoption (messenger message),cautionary (fear message), and a fact-based message as a control message. They were developed based on the consensus by a multidisciplinary team of expert consensus including experts from public health, epidemiology, social and behavioral communication, and cultural anthropology that based on their past experiences of immunization campaigns came to a consensus with a hypothesis that vaccine safety, healthcare workers as messengers, and social norms can have a positive effect on vaccine uptake compared to the fact-based message. According to the experts, positive messaging and the importance of community are cultural values in Ghana, which makes safety and social norm messages potentially effective. Experts also perceived healthcare workers as trusted sources of information for people in Ghana. Fear-based message frame was included as it is often the preferred way to frame messages by risk communication experts [52]. The frames and corresponding nudges can be found in Table 2.

**Table 2:** Message frames and nudges.

Message frame	Nudge
Social norm message	“Most people in your community are getting their children vaccinated against polio. Get your child vaccinated against polio.”
Fear message	“Polio causes paralysis and sometimes death. Get your child vaccinated against polio!”
Safety message	“Polio vaccines are safe. Get your child vaccinated against polio.”
Messenger message	“Polio vaccines are recommended by health professionals. Get your child vaccinated against polio.
Fact-based /control message	“Protect your child against polio. Get your child vaccinated against polio.”

### Sample size

As this was a cross-sectional survey, a proportion (prevalence) approach was used to determine the sample size. The 2014 Ghana Demographic and Health Survey reported 80% complete coverage of polio vaccination for eligible children [53]. By regions, the range is 69–97% (Northern vs. Upper West) [54]. Using the average proportion of full polio vaccination (80%) rate and zoning the country into three clusters, the Dean, Sullivan and Soe (2013) equation was used to estimate a sample of 738 [55].

Following data collection, there were 764 complete responses from caregivers which were further pared down to 708 caregivers after data transformation (see “Data transformation” for more details) which is the final full sample for the analysis in this study. This sample is also further truncated for the analysis of intent to vaccinate (250 caregivers who have not yet vaccinated their children) and the effect of messaging (53 caregivers out of the aforementioned 250 who say they will not vaccinate their children).

### Recruitment and data collection

The respondents were drawn from the mobile phone service database of Viamo’s 321 and Agoo that delivers targeted messages on health, education, and agriculture to the public in Ghana implemented by the GHS, UNICEF, and VIAMO. These are promoted as mobile phone-based services that members of the public can call voluntarily to listen to the messages.

In order to reach active numbers (i.e., phone numbers that are active and can receive calls), all 80,000 contacts were drawn from the database. They were uploaded onto the IVR platform and called in batches of 4,764 given the call capacity of the IVR system at a time. Each respondent within a batch had an equal chance of being contacted to take the survey. The sample included participants from all 16 administrative regions of Ghana. Data collection took place from October to December 2021. The system reached 39,734 unique callers of which 4,404 agreed to participate but only 3,305 callers met the inclusion criteria (mothers having children under 5 years). A total of 764 callers responded to the survey of which 70% responded in Twi, 16% in English, and 11% Ewe. The remaining respondents used Dagbani, Hausa, and Ga languages to respond to the survey. Respondents who missed the call or were unwilling to complete the survey at the time of the call were able to call back by dialing the number on which they missed the call. The system also immediately called them back and gave them the opportunity to complete the survey. Respondents were randomly assigned each to listen to one of the message frames after which they answered the questions relating to the message frames. Given that there were four message frames, four groups were created each of which listened to one message frame.

### Procedures

Upon receiving and answering the automated call to participate in the survey, respondents were asked to choose among the six predominantly spoken languages in Ghana: English, Twi, Ga, Ewe, Dagbani and Hausa. The rest of the survey was presented in the selected language. Respondents were then asked to provide their voluntary consent to participate in the survey, and consent to be contacted for future surveys. Respondents were also asked to verify that they are mothers with children younger than five years old. The survey immediately ended for respondents who did not consent to the survey and/or who did not meet the inclusion criterion (mothers of children younger than five years old).

In part 1 of the survey, respondents were first asked if they ever gave the polio vaccine to their child. If the respondents reported not having given the polio vaccine to their child, they were presented with questions measuring their intentions the to give polio vaccine to their children. If the respondents reported having vaccinated their children with the polio vaccine, they were presented with questions measuring the factors influencing their vaccination behavior. Respondents then rated their perceptions on the various drivers of vaccination by answering yes, no, or I don’t know. Next, respondents were asked to provide demographic information, including education and location (urban/rural).

In part 2 of the survey, respondents were randomly assigned to one of five nudges. After listening, respondents were asked if they would go to vaccinate their children or if they would recommend polio vaccine to others.

### Data transformation

Before estimating our results, we transformed the collected survey data. As mentioned previously, there were complete survey responses from 764 caregiver respondents. This number was further pared down to 708 caregivers after data transformation, giving us our final full sample of caregivers. This is the sample of caregivers that we analyze to see what drives vaccine uptake, i.e., the factors that exert influence on caregivers to vaccinate their children against polio.

This number was arrived upon by first removing all responses for which respondents have indicated that they “prefer not to answer” on key questions (i.e., vaccine uptake, intent to vaccinate, psychological factors, sociological factors and environmental factors). These respondents’ true behavior/preferences are not observed. Therefore, their responses do not lend themselves to inferential analysis. After dropping these responses, our sample of caregivers came down to 708 complete responses.

We also re-coded all responses to make them binary, such that a response indicating “correct” perceptions/knowledge about the nature and severity of polio as a disease, or positive perceptions/knowledge regarding polio vaccines takes a value of 1, and 0 otherwise. This is because in looking for drivers of polio vaccines and for a caregiver’s intention to vaccinate their child, we want to know the extent to which “correct” psychological, sociological, and environmental factors influence our outcomes of interest. Consequently, responses where these factors are not confirmatory (i.e., “no” or “don’t know”) can be bundled together as a single counterfactual. There are two additional practical reasons for the re-coding described above: i) “don’t know” responses do not have a logical interpretation in regression results; and ii) given an already large number of covariates, including “don’t know” response for each of those in the estimation leads to a compromise on degrees of freedom, which hinders statistical inference.

In order to see what drives intent to vaccinate, we assessed data from 250 caregivers from the aforementioned total sample of 708 caregivers. These 250 caregivers are mothers who have not yet vaccinated their children against polio but say that they intend to do so in the future.

Lastly, we further truncated our sample, from the 250 mothers who hadn’t vaccinated their children, to 53 caregivers who say that they have not yet vaccinated their children and do not intend to vaccinate them in future vaccination drives. Using data from these respondents, we assess the potential effect of different kinds of messaging in encouraging these women to vaccinate their children.

### Choice of regression method

Given the categorical nature of our survey responses, the choice of identification strategy for our analysis is between a linear probability model (LPM) estimated using ordinary least squares (OLS), and a logistical regression model. Given our relatively small total sample size, i.e., 708 caregivers, and the lack of variation in responses, our dataset does not lend itself for analysis via a logistical regression. Therefore, we use LPM with OLS estimates for our analysis.

One of the most oft-cited issues with LPM through OLS is that can have results that are biased and inconsistent, and may also have induced heteroskedasticity with binary outcomes like in our study [56]. Crucially, however, such bias is only present when the underlying predicted probabilities in a LPM fall out.

The predicted probabilities underlying the estimates in our regressions do not fall outside the unit interval; this ensures that our estimates are not biased, nor inconsistent—thus avoiding the biggest pitfall of the LPM method. Additionally, our LPM estimates with OLS do not suffer from the issue of induced heteroskedasticity because we use heteroskedasticity robust standard errors. Lastly, due to our OLS estimates, our results are easier to interpret and understand than a LPM without OLS estimates.

In estimating our results, we include controls for different regions. Controls for rural/urban location and respondents’ religion were removed, as including them did not change our results but reduced degrees of freedom. Including these controls, as well as dummy variables, which we have done in our analysis, would also make logistic regression unviable in our study.

### Ethical considerations

The study protocol was approved by the Ghana Health Services Ethical Review Board (GHS-ERC 008/09/21). Voluntary participation was duly addressed. Participants were asked for written consent to participate in the survey. Consent was provided on the first page of the survey and it required participants to click a button indicating consent to participation before proceeding to decide the language in which they would want to take the survey. Participants were assigned unique identifiers to ensure anonymity. To ensure privacy, participants were allowed to skip questions that they found too sensitive. Strict digital information security measures were applied to data storage.

## Results

### Description of the survey respondents for the whole sample

The survey respondents included a total of 708 mothers with children younger than five years old, who came from all 16 administrative regions of Ghana. The Greater Accra region had the largest number of respondents with 23.6% followed by the Ashanti region with 21.3% of respondents. The region with the lowest number of respondents was the Western-North region with 0.6%. Over half of the respondents were from rural areas (58.4%). Over 25% of the participants were young mothers in the age group of 14–17 whereas the great majority (55.4%) belonged to the age group from 18–30 years old. Approximately half of the respondents (50.9%) had a secondary education followed by 32.6% of respondents with primary education and 16.5% with tertiary education. Almost 60% of the participants reported being married; nearly 80% of them (78.1%) had 1–3 children. Only approximately 7% of the respondents (5.6%) had more than seven children. The great majority of the respondents were Christians, whereas 19.1% were Muslims and only 2.8% of the participants belonged to traditional African religions. Overall, 250 (35%) of the respondents had not vaccinated their children against polio and among them, a total of 53 respondents (7.5%) stated that they did not intend to do so. The description of the survey respondents for the whole sample can be found in S2 Table; the table describes the survey participants for the whole sample (N = 708) including gender, age, education, religion, marital status, region, and urban or rural area.

### Factors influencing the uptake of the polio vaccine

To assess vaccine uptake, the outcome variable in our LPM model was a child’s current vaccination status as reported by their caregiver, i.e., a binary variable. Table 3 shows our regression estimates. For these regressions, the full sample of 708 caregivers was utilized. Each column represents a separate regression, and each regression is statistically significant up to the 95% CI; please see S3 Table for the full tables of each regression. The effects of each category of factors within the BDM (i.e., psychological factors, sociological factors, and environmental factors) are displayed separately (columns 1–3 respectively), while the full effects of all three categories of factors are displayed in column 4.

**Table 3:** Linear probability model on psychological, sociological, and environmental factors, as per BDM, that exert influence on whether a child has already been vaccinated against polio (i.e. vaccine uptake).

Variable	Vaccination of children against polio, as influenced by:			
	(1) Psychological Factors	(2) Sociological Factors	(3) Environmental Factors	(4) Overall Model (All three categories of BDM)
Think polio is severe	0.02 (-0.08, 0.12)	-	-	0.01 (-0.09, 0.11)
Think polio causes paralysis	0.17*** (0.06, 0.29)	-	-	0.13** (0.02, 0.24)
Think polio vaccine prevents polio	-0.04 (-0.15, 0.06)	-	-	-0.07 (-0.17, 0.03)
Think polio vaccine is safe	0.20*** (0.10, 0.31)	-	-	0.11** (0.01, 0.22)
Traditional/religious leaders support	-	0.04 (-0.03, 0.12)	-	-0.0004 (-0.07, 0.07)
Healthcare workers support	-	0.19*** (0.10, 0.28)	-	0.11** (0.02, 0.20)
Have HH members who do not support	-	-0.07 (-0.17, 0.02)	-	-0.05 (-0.14, 0.04)
Need permission from HH members	-	-0.12** (-0.22, -0.03)	-	-0.11** (-0.20, -0.01)
Trust healthcare workers	-	0.04 (-0.04, 0.12)	-	0.03 (-0.04, 0.11)
Seen/heard something negative about vaccine	-	-	0.09** (0.02, 0.16)	0.10*** (0.03, 0.17)
Find it difficult to get vaccine	-	-	-0.20*** (-0.27, -0.12)	-0.16*** (-0.23, -0.08)
Vaccinators provide enough information	-	-	0.18*** (0.11, 0.25)	0.12*** (0.05, 0.20)
[Interaction term] Have HH members who don’t support vaccine-Need permission to vaccinate	-	0.03 (-0.12, 0.17)	-	-0.01 (-0.15, 0.13)
Intercept	0.25*** (0.11, 0.39)	0.45*** (0.33, 0.57)	0.49*** (0.39, 0.59)	0.33*** (0.16, 0.50)
Are region controls applied?	Yes	Yes	Yes	Yes
Regression sample size	708	708	708	708
Regression p-value	0.00	0.00	0.00	0.00

95% confidence interval in parentheses.

*p<0.1

**p<0.05

***p<0.01.

In the overall mode (Table 3, column 4), under psychological factors, the regression model showed that respondents who knew that polio causes paralysis were 13% more likely to vaccinate their children compared to our counterfactual, which is all those who either said those who did not vaccinate their children (i.e., a “no” response) or those who were unsure about it (i.e., a “don’t know” response) with statistically significant coefficients of 0.13 (95% CI: 0.02, 0.24). Similarly, people who perceived the polio vaccine as safe were 11% more likely to vaccinate their child than those who did not believe so or who were unsure about it with statistically significant coefficients of 0.11 (95% CI: 0.01, 0.22).

In terms of sociological factors in the overall model, the regression model showed that respondents who perceived support from healthcare workers were 11% more likely to vaccinate their children than those who either did not perceive the support or were not sure about it, with statistically significant coefficients of 0.11 (95% CI: 0.02, 0.20).

All three environmental factors measured in this study were found to be statistically significant predictors for polio vaccine uptake—both in the overall model (Table 3, column 4) and also on their own (Table 3, column 3). This means that those who found it difficult to obtain the polio vaccine were 16% less likely to have vaccinated their child—with a statistically significant coefficient of -0.16 (95% CI: -0.23, -0.08)—while those who were satisfied with vaccinator-provided information were 12% more likely to have vaccinated their child—with a statistically significant coefficient of 0.12 (95% CI: 0.05, 0.20). Notably, the regression showed that those who had seen or heard something negative about the polio vaccine were 10% more likely—with a statistically significant coefficient of 0.10 (95% CI: 0.03, 0.17)—to have vaccinated their child than those who had not heard anything negative about the polio vaccine.

The geographical location of the respondents was not found to influence the uptake of the polio vaccine with the exception of regions of Greater Accra and Volta where caregivers were 20% more likely and 36% more likely, respectively, than caregivers in the region of Ashanti to have already vaccinated their children against polio; the respective coefficients were statistically significant 0.20 (95% CI: 0.09, 0.30) for Greater Accra and 0.36 (95% CI: 0.25, 0.48) for Volta. Those who were Muslims (N = 134) were 11% less likely to vaccinate their children than those who were Christians (N = 533), with a statistically significant coefficient of -0.11 (95% CI: -0.21, -0.02).

### Factors influencing intention to vaccinate children against polio

We assessed the intention to vaccinate children against polio in our sample of caregivers in a similar manner to vaccine uptake: intent to vaccinate was taken as a binary outcome variable, while the effects of individual components of the BDM were regressed separately and then all together. For these four regressions, the sample was limited to only 250 caregivers who had not yet vaccinated their children against polio, but said that they intended to vaccinate them in the future.

Table 4 shows our results for the regressions for intent to vaccinate, with columns 1–3 displaying results for psychological factors, sociological factors and environmental factors of BDM, respectively; column 4 shows the full effects of all three category of factors. In this table as well, each column represents a separate regression, and each of these regressions is statistically significant up to the 95% CI. Please see S4 Table for the full tables of each regression.

**Table 4:** Linear probability model on psychological, sociological, and environmental factors for intention to give polio vaccine to children.

Variable	Intent to vaccinate children against polio, as influenced by:			
	(1) Psychological Factors	(2) Sociological Factors	(3) Environmental Factors	(4) Overall Model (All three categories of BDM)
Think polio is severe	0.01 (-0.11, 0.13)	-	-	-0.01 (-0.13, 0.11)
Think polio causes paralysis	-0.02 (-0.15, 0.11)	-	-	-0.02 (-0.16, 0.11)
Think polio vaccine prevents polio	0.05 (-0.11, 0.20)	-	-	0.04 (-0.12, 0.20)
Think polio vaccine is safe	0.19** (0.04, 0.33)	-	-	0.12 (-0.03, 0.27)
Traditional/religious leaders support	-	0.05 (-0.06, 0.16)	-	0.01 (-0.09, 0.12)
Healthcare workers support	-	0.17*** (0.06, 0.28)	-	0.12** (0.01, 0.24)
Have HH members who do not support	-	0.10 (-0.05, 0.25)	-	0.07 (-0.07, 0.22)
Need permission from HH members	-	-0.004 (-0.16, 0.15)	-	-0.02 (-0.16, 0.13)
Trust healthcare workers	-	0.04 (-0.07, 0.15)	-	0.03 (-0.08, 0.14)
Seen/heard something negative about vaccine	-	-	-0.01 (-0.12, 0.10)	-0.02 (-0.13, 0.09)
Find it difficult to get vaccine	-	-	-0.07 (-0.16, 0.03)	-0.07 (-0.16, 0.03)
Vaccinators provide enough information	-	-	0.19*** (0.09, 0.29)	0.12** (0.01, 0.23)
[Interaction term] Have HH members who don’t support vaccine-Need permission to vaccinate	-	-0.11 (-0.32, 0.11)	-	-0.07 (-0.28, 0.14)
Intercept	0.57*** (0.39, 0.75)	0.56*** (0.37, 0.76)	0.69*** (0.56, 0.82)	0.53*** (0.30, 0.76)
Are region controls applied?	Yes	Yes	Yes	Yes
Regression sample size	250	250	250	250
Regression p-value	0.00	0.00	0.00	0.00

95% confidence interval in parentheses.

*p<0.1

**p<0.05

***p<0.01.

Out of all the caregivers who had not yet vaccinated their child (N = 250, 35% of the full sample), the majority, i.e., 28% of the full sample of 708 caregivers, expressed an intent to vaccinate their children in the next polio campaign. In the overall regression model (Table 4, column 4), we see that one sociological factor—receiving support from healthcare workers (“Healthcare workers support”)—was found to have statistically significant effect on the intention of a caregiver to take vaccinate their child in the future, with a coefficient of 0.12 (95% CI: 0.01, 0.24). Likewise, one environmental factor—being satisfied with the information provided by vaccinators (“Vaccinators provide enough information”)—was found to be statistically significant with a coefficient of 0.12 (95% CI: 0.01, 0.23). This means that those who received support from health workers and those who felt satisfied with the information provided by vaccinators were each 12% more likely to intend to vaccinate their child in the next polio vaccination campaign. In the analysis of psychological factors, thinking the polio vaccine is safe had a statistically significant coefficient of 0.19 (95% CI: 0.04, 0.33), but this was not significant in the overall regression.

Similar to vaccine uptake, geographical location did not influence caregivers’ intention to vaccinate their children, except in the Brong Ahafo and Northern regions, where respondents were 23% and 19% more likely, respectively, to intend to vaccinate their child in the next polio vaccination campaign, compared to caregivers in Ashanti; the respective coefficients were 0.23 (95% CI: 0.04, 0.42) for Brong Ahafo and 0.19 (95% CI: -0.002, 0.38) for Northern. Respondents who reported their religion as Traditional African were 31% less likely to intend to vaccinate their children, with a statistically significant coefficient of -0.31 (95% CI: -0.64, 0.03) than those who reported being Muslims or Christians.

### The effectiveness of messaging frames

The effectiveness of messaging frames was measured in the full sample (N = 708) by the proportion of respondents reporting their intention to give the polio vaccine to their child or intention to recommend the polio vaccine to others. Table 5 shows the results of this assessment, which helps us understand how effective messaging can be in encouraging (i.e. “nudging”) people to vaccinate their children against polio. Each messaging frame’s effectiveness is assessed in comparison to the control message (“Protect your child against polio. Get your child vaccinated against polio!”). Columns 1 of Table 5 shows the results of messaging on the full sample (N = 708), of caregivers’ intention to vaccinate a child; column 2’s results also show the effect of messaging on intent to vaccinate on the entire sample (N = 708), but controlling for whether a caregiver’s child was already vaccinated or a caregiver was intending to vaccinate their child/children before the message was heard by the caregiver; in column 3 and 4, the outcome variable is a caregiver’s willingness to recommend polio vaccination to others; column 3 is on the entire sample (N = 708), and column 4 is also on the entire sample (N = 708) but controlling for whether already vaccinated or intending to vaccinate before the message played. Columns 1, 2 and 4 are statistically significant at a 95% CI. Please see S5 Table for the full tables of each regression in Table 5.

**Table 5:** Efficiency of the nudges with different message frames on the full sample of 708 caregivers.

Variable	Effect on intention to vaccinate a child in future		Effect on willingness to recommend vaccination to others	
	(1)	(2)	(3)	(4)
Social Norms Message	0.05* (-0.01, 0.12)	0.06* (-0.004, 0.12)	-0.02 (-0.08, 0.04)	-0.02 (-0.08, 0.04)
Fear Message	0.02 (-0.05, 0.08)	0.02 (-0.04, 0.08)	0.01 (-0.05, 0.06)	-0.01 (-0.04, 0.06)
Safety Message	0.03 (-0.04, 0.09)	0.02 (-0.04, 0.08	-0.02 (-0.08, 0.03)	-0.02 (-0.07, 0.03)
Messenger Message	0.03 (-0.03, 0.10)	0.03 (-0.03, 0.10)	-0.01 (-0.06, 0.05)	-0.01 (-0.06, 0.04)
Child has received polio vaccine	-	0.02 (-0.02, 0.06)	-	0.05** (0.004, 0.09)
Will vaccinate child in next campaign	-	0.16*** (0.08, 0.24)	-	0.15*** (0.07, 0.23)
Intercept	0.90*** (0.85, 0.95)	0.75*** (0.65, 0.85)	0.95*** (0.91, 0.98)	0.79*** (0.70, 0.88)
Regression sample size	708	708	708	708
Regression p-value	0.47	0.01	0.82	0.00

95% confidence interval in parentheses.

*p<0.1

**p<0.05

***p<0.01.

Messaging frame coefficients are relative to the “control” messaging frame.

In column 2 of Table 5, only the social norms frame was statistically significant with a coefficient of 0.06 (95% CI: -0.004, 012), along with a control for “will vaccinate child in next campaign” which was statistically significant at 0.16 (95% CI: 0.08, 0.24). In other words, when considering the effectiveness of messaging in the full sample of caregivers (N = 708), messaging does not appear to affect intention to vaccinate. This is consistent with the finding that, out of those who say that their children are not vaccinated (N = 250), a majority (N = 197) of caregivers say that they already intend to vaccinate their children. Additionally, in column 4 of Table 5, no message frame was effective at compelling caregivers to recommend the polio vaccine to others; only those who had already vaccinated their children or those who had not vaccinated their children but were intending to vaccinate them appeared more likely to recommend vaccination to others. The respective coefficients were statistically significant at 0.05 (95% CI: 0.004, 0.09) for caregivers who had already vaccinated their children and 0.15 (95% CI: 0.07, 0.23) for those who had not vaccinated their children but were intending to vaccinate them.

Following from this, in order to assess the effectiveness of nudge messages against vaccine hesitancy, the nudges were also tested in a truncated sample of those caregivers who had not vaccinated their children and also stated that they do not intend to do so in the future (N = 53).

Table 6 shows the results of this assessment. Here too, each messaging frame’s effectiveness is assessed in comparison to the control message (“Protect your child against polio. Get your child vaccinated against polio!”). Column 1 in Table 6 shows the results of messaging on vaccine-hesitant caregivers’ (N = 53) intention to vaccinate their kids in the future, while column 2 in Table 6 shows the results of messaging on the same sample of vaccine hesitant caregivers’ (N = 53) willingness to recommend vaccination to other people. Only column 1 in Table 6 is statistically significant at a 95% CI. Please see S6 Table for the full tables of each regression in Table 6.

**Table 6:** Efficiency of the nudges with different message frames on the truncated sample of 53 vaccine-hesitant caregivers.

Variable	Intention to vaccinate a child	Willing to recommend vaccination to others
	(1)	(2)
Social Norms Message	0.45** (0.10, 0.81)	0.27 (-0.12, 0.66)
Fear Message	0.13 (-0.30, 0.55)	0.37** (0.02, 0.72)
Safety Message	0.45** (0.10, 0.81)	0.27 (-0.12, 0.66)
Messenger Message	0.42** (0.03, 0.81)	0.20 (-0.24, 0.65)
Child has received polio vaccine	-	-
Will vaccinate child in next campaign	-	-
Intercept	0.45*** (0.15, 0.76)	0.55*** (0.24, 0.85)
Regression sample size	53	53
Regression p-value	0.05	0.35

95% confidence interval in parentheses.

*p<0.1

**p<0.05

***p<0.01.

Messaging frame coefficients are relative to the “control” messaging frame.

In the truncated sample of vaccine-hesitant caregivers (N = 53) the social norm frame, safety frame, and messenger frames, when compared against the control message, were all similar in their effectiveness. Both social norms frame (“Most people in your community are getting their children vaccinated against polio. Get your child vaccinated against polio”) and safety frame (“Polio vaccines are safe. Get your child vaccinated against polio”) were 45% more likely than the control message to make respondents report intent to give polio vaccine to their child than those hearing the control frame (“Protect your child against polio. Get your child vaccinated against polio.”).Those who heard the messenger frame (“Polio vaccines are recommended by health professionals. Get your child vaccinated against polio!”) were 42% more likely to get their child vaccinated than respondents who heard the control frame. Respectively, they had coefficients of 0.45 (95% CI: 0.10, 0.81), 0.45 (95% CI: 0.10, 0.81), and 0.42 (95% CI: 0.03, 0.81).

Additionally, in the aforementioned truncated sample (N = 53), only the fear message frame (“Polio causes paralysis and sometimes death. Get your child vaccinated against polio!”) appears to have an effect in predicting whether a respondent would recommend the polio vaccine to others. It has a statistically significant coefficient of 0.37 (95% CI: 0.02, 0.72), which means that the fear message was 37% more likely than the fact-based control message to compel people to recommend vaccination to others.

## Discussion

This study provided valuable insights into the facilitators and barriers that influence the decision of mothers to give polio vaccines to their children younger than 5 years in Ghana, with possible implications for other similar low and middle-income countries. The facilitators included the understanding that polio can cause paralysis and confidence in vaccine safety, perceived support from healthcare workers to take the vaccine, satisfaction with the provision of information provided by vaccinators, and having heard of negative aspects of vaccination. The barriers included difficulties accessing vaccination and the need for permission from a male family member to vaccinate children. The study also identified factors that can encourage vaccine uptake among those who still have not vaccinated their children including perceived support from healthcare workers and satisfaction regarding polio and vaccine-related information provided by vaccinators. Lastly, the study showed that various message frames were statistically significant although they did not affect behavior.

The findings indicate that vaccine demand creation in Ghana should focus on communicating the safety of the vaccine as well as the negative consequences of poliovirus; namely paralysis. The safety of the vaccines is a common factor that has been identified as influencing polio vaccine uptake in a number of countries such as India, Nigeria, and Pakistan [57–59]. Having heard of negative aspects of the vaccine was also identified as a facilitator for behavior. Further qualitative investigations are needed to understand how this influences the behavior and how this information can be utilized in polio vaccine demand creation. Meanwhile, it is important to keep monitoring polio-related rumors that may be circulating in communities to be aware of the ongoing conversations and to be able to intervene when misinformation is spreading [60]. This highlights the importance of having social listening in place. Ghana Health Services together with the UNICEF country office have established a misinformation management task force for COVID-19 misinformation management that could be expanded to include polio-related rumors and misinformation [61].

A notable number of caregivers experience logistical difficulties in vaccinating their child. It would be of utmost importance to understand what factors make it difficult for the mothers in Ghana where polio vaccine campaigns are also predominantly house-to-house based, which have been evaluated as the most cost-effective strategy in other countries in the world such as Egypt, Ethiopia, and India to achieve universal coverage and thus to eradicate polio [62]. In India and Ethiopia, mothers perceived house-to-house visits positively as they were based on an interactive counseling approach and they were prepared to respond to specific questions about the disease and the vaccine as well as to provide logistical and practical information. In both countries house to house visits were designed based on the context and the concerns of the community. For example, in India, community health workers received training on how the vaccine was produced to reassure families that the vaccine contained no ingredients that violated Muslim religious requirements, whereas, in Ethiopia, contents addressed specific traditional beliefs about the spiritual rather than biomedical aetiology of paralysis [63].

The findings of this study also revealed that difficulties to access the vaccine were considered as a barrier to taking the polio vaccine, which is interesting as polio campaigns in Ghana have been typically implemented by vaccinators making house-to-house visits. It would be important to investigate what kind of logistical problems are linked with house-to-house vaccination including the quality of the visits. The study also pointed out that mothers perceived the permission of a male member of their family to vaccinate their children as a barrier, which highlights the need to include male members of the family in the vaccine demand activities. Gender-based challenges in polio programming have been documented in particular related to gender power relations in many countries in the world including India, Pakistan, Afghanistan, Ethiopia, Bangladesh, the Democratic Republic of the Congo, Indonesia, and Nigeria [64]. Parental counseling has been suggested as an intervention to engage male members of families in vaccine demand creation [65]. In addition, evidence-based and multi-faceted communication campaigns that take a whole society approach have been found effective in addressing male members of families with polio messaging [66].

The study highlighted the central role of healthcare workers in vaccine demand generation in Ghana in which healthcare workers appear to be strongly preferred and trusted information sources for polio and vaccine-related information. Vaccination is an opportunity for mothers to get in touch with trusted sources of information, namely healthcare workers. Lack of access to the vaccines on the other hand may result in the dependency on other sources of information, such as community leaders or broadcast media, which the study results suggest influence vaccine uptake negatively. Healthcare workers can positively influence behavior and intention to take the vaccine This aligns with many global studies that show that healthcare workers are a cornerstone of vaccine-related communication [27,28]. The interaction between patients and healthcare providers is known to be of utmost importance in maintaining confidence in vaccination [29] highlighting the importance of engaging healthcare providers in polio communication. Likewise, it is important to ensure that they are well informed and have communication skills to deliver messages appropriately. Efforts should be made to build the capacity of healthcare workers to communicate polio and vaccine-related messages. Polio vaccines are best communicated when parents and healthcare staff have the opportunity to openly discuss the prevention of infectious diseases and vaccines. Far too often parents remain unsatisfied with the amount of information they receive in brief physician encounters, highlighting the need to identify context-specific channels to discuss and communicate safety and institutionalize communication during patient-provider encounters. Standard operating procedures, developed in co-creation with healthcare workers to ensure acceptability and practicality, should be considered [30,31].

Our study was not able to identify one specific message frame that influenced the intention to take the vaccine or recommend the vaccine to others. It is possible that the sample size was too small to detect these differences. It is also possible that the survey that preceded the message nudges biased the views of the respondents. In future experiments, it may be worthwhile to run the survey and the experiment separately. However, our findings indicate that all the frames had a positive impact and should be therefore be considered and further explored. The use of a norm-based frame can be a good fit in particular to the cultural context of Ghana, in which people emphasize communal values such as family, respect for the elderly, and honor in traditional rulers [32]. Several studies conducted in other countries confirm that social norm framed messages have a positive impact on vaccine uptake [34,35] including studies that have explored polio vaccine [36,37]. Future research could test different types of social norm messages e.g., mothers like you are increasingly vaccinating their children. It is worth noting that social norms may also have a negative effect on behavior because the perception that “not everyone is doing it” can decrease the intention to act. Therefore, one has to be careful not to send out messages that people are not vaccinating children, as this may lead to unintended effects of vaccine hesitancy [39,40]. The safety frame also requires further consideration as safety was identified as a topic that encourages the uptake of vaccines; it is likely that safety as a message frame has utility.

This study is to our knowledge the first mobile phone-based survey in Ghana using Random Digit Dialing (RDD) to recruit study participants. The use of an automated mobile phone-administered survey based on voice response (IVR system) generally worked well. For example, the callback feature of the system allowed participants to take the survey at their convenience. However, there were limitations. As a mobile phone-based data collection, it left out those who do have access to such devices. Although Ghana has the highest mobile penetration in West Africa, data shows that women in low and middle-income countries are 40% less likely to use a cell phone with internet access than men. These women are often the most vulnerable ones in society [45]. Field-based face-to-face surveys could be conducted in the future to compensate for the gap. The use of a mobile phone-based database linked with participation in voluntary health promotion activities may create further bias as respondents who belong to the databases may be those that are particularly interested in health-related topics. Accordingly, they may be less hesitant towards the polio vaccine. As polio vaccine coverage is high in Ghana, with 93% of children being vaccinated [2], the identification of those who have not been vaccinated may require more targeted recruitment strategies. Mobile based surveys can include only a limited number of questions that makes the inclusion of different types of variables restricted. Accordingly, the findings need to be considered carefully taking into consideration that many variables that may have an impact on the intention of mothers to vaccinate their children at such a socioeconomic level are not included in the study.

## Conclusions

The findings from this study suggest that most women with children under the age of 5 appear to have vaccinated their children against polio. Many more caregivers express an intention to vaccinate their children, never having done so before. The behavior and the intention to vaccinate are both driven by a number of factors that must be addressed to create demand for the polio vaccine. More research is required to understand the impact of different message frames on vaccine behavior and intention to vaccine children.

## Supporting information

S1 Table Survey questionnaire. (PDF)

S2 Table Description of the survey respondents for the whole sample. (DOCX)

S3 Table Details of regressions in Table 3. (DOCX)

S4 Table Details of regressions in Table 4. (DOCX)

S5 Table Details of regressions in Table 5. (DOCX)

S6 Table Details of regressions in Table 6. (DOCX)

S1 Dataset (CSV)
