# Supplementary material for: Strengthening polio vaccine demand in Ghana: Understanding the factors influencing uptake of the vaccine and the effectiveness of different message frames
Source: PLoS One. 2023 Feb 10;18(2):e0279809. doi: 10.1371/journal.pone.0279809 (PMC9916648; doi:10.1371/journal.pone.0279809)
Supplement: S1 Table — (PDF) [file pone.0279809.s001.pdf]

UNICEF Polio Survey Instrument

Mothers of Children under 5 years

Introduction

Hello! This call is from Ghana Health Service and UNICEF. This call is to help Ghana Health Service and UNICEF better understand the things that affect the vaccinationation of your child against Polio. This understanding will help Ghana Health Service and UNICEF to improve the well being of children in the country so your opinion is very important to us! Your participation is voluntary. You may choose to skip any question or discontinue the survey at any time for any reason. Any information you provide will not be disclosed to anyone and would be stored in a secure data file system. This survey will last for only 8 minutes.

Consent
Do you agree to take part in this survey?
If you agree to take part in the survey, Press 1
If you do not agree to take part in the survey, Press 2
To listen to the question again Press 0

Gender
Are you male or female?
If you are female, Press 1
If you are male Press 2
To listen to the question again Press 0

Status
Do you have a child or do you take care of a child who is less than 5 years old?
If you have a child who is less than 5 years, Press 1
If you do not have a child who is less than 5 years, Press 2
If you prefer not to answer Press 3
To listen to the question again, Press 0

1. **Have you given your child a polio vaccine before?**
   If your child has ever received polio vaccine, Press 1
   If your child has never received polio vaccine, Press 2
   If you do not know, Press 3
   If you prefer not not answer, Press 4
   To listen to the question again, Press 0

2. **Are you concerned that any of your children may get sick with polio this year?**
   If Yes, you are concerned Press 1
   If No, you are not concerned Press 2
   If you prefer not to answer Press 3
   To listen to the question again, Press 0
3. **Do you think polio is severe?**
   If you think polio is severe Press 1
   If you think polio is not severe Press 2
   If you do not know, Press 3
   To listen to this question again, Press 0

4. **Do you think polio causes paralysis?**

If you think Polio causes paralysis Press 1
If you think Polio does not cause paralysis , Press 2
If you do not know Press 3
To listen to the question again, Press 0

5. **Do you think polio vaccines prevent polio?**
   If you think polio vaccines prevent polio, Press 1
   If you think polio vaccines do not prevent polio, Press 2
   If you are not sure Press 3
   To listen to the question again, Press 0

6. **Do you think Polio vaccine is safe?**
   If you think Polio Vaccine is safe, Press 1
   If , you think Polio Vaccine is not safe Press 2
   If you do not know Press 3
   To listen to the question again, Press 0

7. **Do you think getting a polio vaccine will be important for the health of your child under 5 years?**
   If you think getting polio vaccine for your child is important Press 1
   If you think getting polio vaccine for your child is not important Press 3
   If you do not know, Press 4
   To listen to this question again, Press 0

8. **Have you seen or heard anything negative about polio vaccines?**
   If you have seen or heard something negative about polio vaccine Press 1
   If you have not seen or heard anything negative about polio vaccine Press 2
   If you do not know Press 3
   If you prefer not to answer Press 4
   To listen to the question again, Press 0

9. **Do you find it is difficult or easy to get polio vaccination for your child?**
   If you find it easy to get polio vaccination for your child, Press 1
   If you find it , easy to get polio vaccination for your child Press 2
   If you are not sure Press 3
   To listen to the question again, Press 0
10. **Which one of the following is the main challenge that makes it hard for you to get your child or children vaccinated? Please listen to all options before you answer.**
    If it is because your house and where you get vaccination is too far Press 1
    If it is because the times the places are opened for vaccination are not convenient Press 2
    If it is because you think there is cost involved in vaccination Press 3
    If it is because the waiting time during vaccination is too long, Press 4
    If is because some people are turned away Press 5
    If you do not know Press 6
    To listen to the question again, Press 0

11. **Do traditional/religious leaders in your community support children receiving polio vaccines?**
    f Yes, traditional/religious leaders support Press 1
    If No, traditional/religious leaders do not support Press 2
    If you prefer not to answer Press 3
    If you do not know Press 4
    To listen to the question again, Press 0

12. Do healthcare workers in your local health center support caregivers to get their children vaccinated against polio??

If healthcare workers support mothers to get polio vaccine for their children Press 1
If healthcare workers do not support mothers to get polio vaccine for their children, Press 2
If you prefer not to answer Press 3
If you do not know Press 4
To listen to the question again, Press 0

13. Do you have household members who do not support children to receive polio vaccine?
If you have household members who do not support polio vaccination Press 1
If you have household members who support polio vaccination Press 2, Press 2
If you prefer not to answer Press 3
If you do not know Press 4
To listen to the question again, Press 0

14. If it was time for your child to get a polio vaccination, would you need permission from your family members to go and vaccinate your child?
If you will need permission from your family members, Press 1
If you will not need permission from any family member Press 2
If you prefer not to answer Press 3
If you do not know Press 3
To listen to the question again, Press 0

15. **Based on your previous experience do you feel the vaccinators provide enough information about polio vaccination to you?**
If vaccinators provided enough information Press 1
If vaccinators did not provide enough information Press 2
If vaccinators did not provide any information at all Press 3
If you prefer not to answer Press 4
If you do not know Press 5
To listen to the question again, Press 0

16. How much do you trust the health care providers who would give your child a polio vaccine?
If you do not trust them at all Press 1
If you just trust them Press 2
If you trust them very much Press 3
If you prefer not to answer Press 4

17. Will you accept to give polio vaccine for your child during the next polio campaign?
If Yes, Press 1
If No, Press 2
If you prefer not to answer Press 3
If you are not sure Press 4
To listen to the question again, Press 0

18. Which of the following is the main reason why you would not give polio vaccine to your child during the next polio campaign?
If the fear of side effect is the main reason, Press 1
If it is because you think polio is not dangerous Press 2

If it is because you think your child has received enough polio vaccine already Press 3
If it is because of some other reason which is not mentioned Press 4
If you prefer not to answer Press 5
If you do not know Press 6
To listen to the question again, Press 0

19. **Which one of the following do you consider as the most important information that you would need on polio vaccination?**

If you consider information on safety of the polio vaccine as most important Press 1
If you consider recommendations from health workers as most important, Press 2
If you consider recommendation from religious or traditional leaders as most important, Press 3
If you do not know Press 4
To listen to the question again, Press 0

20. **Which one of the following is the main source from which you receive health information?**
It is medical doctors, nurses, or other health service providers Press 1
If it is community and religious leaders, Press 2
If it is TV, radio and newspaper Press 3
If it is community information center Press 4
If it is social media Press 5
If you prefer not to answer Press 6
If you do not have Press 7
To listen to the question again, Press 0

Behaviour Change Message Frames
Great job so far! We are almost done. Listen to the following short messages and answer the short questions that follow. Thank you.

Social Norm
Most people in your community are getting their children vaccinated against polio. Get your child vaccinated against polio!

Fear
Polio causes paralysis and sometimes death. Get your child vaccinated against polio!

Safety
Polio vaccines are safe. Get your child vaccinated against polio!

Messenger
Polio vaccines are recommended by health professionals. Get your child vaccinated against polio!

Control
Protect your child against polio. Get your child vaccinated against polio!

Question 1
After hearing this message, would you take your child for polio vaccination?
If Yes, Press 1
If No, Press 2
If you prefer not to answer Press 3

If you are not sure Press 4
to listen to this question again, Press 0

Question 2

After hearing this message, would you advise other families to take their children for polio vaccination?

If Yes, Press 1
If No, Press 2
If you are not sure Press 3
To listen to the question again, Press 0

Age

How old are you?

If you are within the age range 14 to 17 years, Press 1
If you are within the age range 18 to 30 years, Press 2
If you are within the age range 31 and 45 years, Press 3
If you are above 45 years, Press 4
To listen to the question again, Press 0

Child

How many children do you have?

If you have 1 to 3 children Press 1
If you have 4 to 6 children Press 2
If you have 7 or more children Press 3
If you prefer not to answer Press 4

Region

Which region do you live in?

If you live in the Greater Accra Region Press 1
If you live in the Ashanti Region Press 2
If you live in the Eastern Region Press 3
If you live in the Central Region Press 4
If you live in the Upper West Region Press 5
If you live in the Upper East Region Press 6
If you live in the old Volta Region Press 7
If you live in the old Western Region Press 8
If you live in the old Brong Ahafo Region Press 9
If you live in the old Northern Region Press 0

Volta 1

Which of the two regions which were created out of the old Volta Region do you live in?
If you live in the Oti Region Press 1
If you live in the Volta Region Press 2
To listen to this question again, Press 0

Western 1

Which of the two regions which were created out of the old Western Region do you live in?
If you live in the Western-North Region Press 1
If you live in the Western Region Press 2
To listen to this question again, Press 0

Brong Ahafo region1
Which of the three regions which were created out of the old Brong Ahafo Region do you live in?
If you live in the Bono Region Press 1
If you live in the Ahafo Region Press 2
If you live in the Bono East Region Press 3
To listen to this question again, Press 0

Northern 1
Which of the three regions which were created out of the old Northern Region do you live in?
If you live in the Savannah Region Press 1
If you live in the North-East Region Press 2
If you live in the Northern Region Press 3
To listen to this question again Press 0

Religion
Which religion do you belong to?
If you belong to the Christian Religion Press 1
If you belong to the Islamic Religion Press 2
If you belong to the Traditional African Religion Press 3
If you belong to a religion that is not mentioned here Press 4
If you prefer not to answer Press 5
To listen to the question again, Press 0

Location
Where do you live?
If you live in an urban area Press 1
If you live in a rural area Press 2
If you prefer not to answer Press 3
If you do not know Press 4
To listen to the question again, Press 0

Education
What is your level of education?
If you have primary education Press 1
If you have secondary school education Press 2
If you have tertiary education Press 3
If you prefer not to answer Press 4
If you do not know Press 5
To listen to the question again, Press 0

Marital Status
Are you married?
If you are married, Press 1
If you are not married, Press 2
If you prefer not to answer Press 3
To listen to this question again, Press 0
