# Supplementary material for: Strengthening polio vaccine demand in Ghana: Understanding the factors influencing uptake of the vaccine and the effectiveness of different message frames
Source: PLoS One. 2023 Feb 10;18(2):e0279809. doi: 10.1371/journal.pone.0279809 (PMC9916648; doi:10.1371/journal.pone.0279809)
Supplement: S2 Table — (DOCX) [file pone.0279809.s002.docx]

SI_Table 3: Description of the survey respondents for the whole sample

| Demographic variable | Frequency (N) | Percentage (%) |
| --- | --- | --- |
| Rural | 396 | 58.41 |
| Urban | 282 | 41.59 |
| Christian | 533 | 75.82 |
| Muslim | 134 | 19.06 |
| Traditional African | 20 | 2.84 |
| Other | 16 | 2.28 |
| Greater Accra | 167 | 23.6% |
| Ashanti Region | 151 | 21.3% |
| Central Region | 64 | 9.0% |
| Eastern Region | 61 | 8.6% |
| Volta Region | 55 | 7.8% |
| Bono Region | 39 | 5.5% |
| Upper West | 36 | 5.1% |
| North Region | 35 | 4.9% |
| Upper East | 30 | 4.2% |
| North East Region | 20 | 2.8% |
| Ahafo Region | 17 | 2.4% |
| Oti Region | 9 | 1.3% |
| Bono East Region | 7 | 1.0% |
| Savannah Region | 7 | 1.0% |
| Western Region | 6 | 0.8% |
| Western-North Region | 4 | 0.6% |
| 14 - 17 years | 187 | 26.4% |
| 18 - 30 | 392 | 55.4% |
| 31 - 45 | 107 | 15.1% |
| Above 45 years | 22 | 3.1% |
| 1 - 3 children | 529 | 78.1% |
| 4 - 6 children | 110 | 16.2% |
| 7 or more | 38 | 5.6% |
| Primary Education | 220 | 32.6% |
| Secondary Education | 343 | 50.9% |
| Tertiary Education | 111 | 16.5% |
| Married | 400 | 58.7% |
| Not married | 281 | 41.3% |
