# Supplementary material for: Strengthening polio vaccine demand in Ghana: Understanding the factors influencing uptake of the vaccine and the effectiveness of different message frames
Source: PLoS One. 2023 Feb 10;18(2):e0279809. doi: 10.1371/journal.pone.0279809 (PMC9916648; doi:10.1371/journal.pone.0279809)
Supplement: S3 Table — (DOCX) [file pone.0279809.s003.docx]

**S3 Tables: Details of Regressions in Table 3**

**Table 3 Column 1: Psychological Factors**

| **Variable** | **Estimate** | **Std. Error** | **t-value** | **P-value** | **95% CI**  **LL UL** | | **D.F** |
| --- | --- | --- | --- | --- | --- | --- | --- |
| Intercept | 0.24686 | 0.07137 | 3.4590 | 5.753e-04 | 0.106740 | 0.38699 | 694 |
| Think polio is severe | 0.01884 | 0.05270 | 0.3574 | 7.209e-01 | -0.084637 | 0.12231 | 694 |
| Think polio causes paralysis | 0.17057 | 0.05885 | 2.8982 | 3.872e-03 | 0.055015 | 0.28612 | 694 |
| Think polio vaccine prevents polio | -0.04366 | 0.05470 | -0.7981 | 4.251e-01 | -0.151053 | 0.06374 | 694 |
| Think polio vaccine is safe | 0.20236 | 0.05311 | 3.8103 | 1.511e-04 | 0.098086 | 0.30663 | 694 |
| Region Control: Brong Ahafo | 0.09550 | 0.07237 | 1.3195 | 1.874e-01 | -0.046602 | 0.23760 | 694 |
| Region Control: Central | 0.09167 | 0.06920 | 1.3247 | 1.857e-01 | -0.044201 | 0.22755 | 694 |
| Region Control: Eastern | 0.12934 | 0.07066 | 1.8305 | 6.760e-02 | -0.009387 | 0.26808 | 694 |
| Region Control: Greater Accra | 0.18080 | 0.05292 | 3.4167 | 6.709e-04 | 0.076905 | 0.28470 | 694 |
| Region Control: Northern | 0.03574 | 0.07315 | 0.4885 | 6.254e-01 | -0.107893 | 0.17936 | 694 |
| Region Control: Upper East | 0.17548 | 0.09257 | 1.8956 | 5.843e-02 | -0.006276 | 0.35723 | 694 |
| Region Control: Upper West | -0.02516 | 0.09425 | -0.2669 | 7.896e-01 | -0.210202 | 0.15989 | 694 |
| Region Control: Volta | 0.31555 | 0.05695 | 5.5408 | 4.278e-08 | 0.203731 | 0.42736 | 694 |
| Region Control: Western | 0.05501 | 0.16505 | 0.3333 | 7.390e-01 | -0.269044 | 0.37907 | 694 |

*Note*. N = 708, multiple R^2^ = 0.09525, adjusted R^2^ = 0.07831, model p-value = 2.552e-12, CI = Confidence Interval, LL = Lower Limit, UL = Upper Limit, D.F = Degrees of freedom.

**Table 3 Column 2: Sociological Factors**

| **Variable** | **Estimate** | **Std. Error** | **t-value** | **P-value** | **95% CI**  **LL UL** | | **D.F** |
| --- | --- | --- | --- | --- | --- | --- | --- |
| Intercept | 0.44806 | 0.06245 | 7.1745 | 1.872e-12 | 0.32544 | 0.57068 | 692 |
| Traditional/religious leaders support | 0.04388 | 0.03808 | 1.1521 | 2.497e-01 | -0.03090 | 0.57068 | 692 |
| Healthcare workers support | 0.18763 | 0.04568 | 4.1074 | 4.480e-05 | 0.09794 | 0.11865 | 692 |
| Have HH members who do not support | -0.07311 | 0.04914 | -1.4879 | 1.372e-01 | -0.16959 | 0.27732 | 692 |
| Need permission from HH members | -0.12396 | 0.04896 | -2.5316 | 1.157e-02 | -0.22009 | 0.02336 | 692 |
| Trust healthcare workers | 0.03731 | 0.03989 | 0.9353 | 3.499e-01 | -0.04101 | -0.02782 | 692 |
| [Interaction term] Have HH members who don’t support vaccine-Need permission to vaccinate | 0.02924 | 0.07413 | 0.3944 | 6.934e-01 | -0.11631 | 0.17479 | 692 |
| Region Control: Brong Ahafo | 0.04883 | 0.07417 | 0.6583 | 5.106e-01 | -0.09680 | 0.19445 | 692 |
| Region Control: Central | 0.04861 | 0.07006 | 0.6938 | 4.880e-01 | -0.08895 | 0.18618 | 692 |
| Region Control: Eastern | 0.07633 | 0.06987 | 1.0925 | 2.750e-01 | -0.06085 | 0.21352 | 692 |
| Region Control: Greater Accra | 0.16886 | 0.05279 | 3.1986 | 1.444e-03 | 0.06521 | 0.27252 | 692 |
| Region Control: Northern | 0.02482 | 0.07494 | 0.3312 | 7.406e-01 | -0.12231 | 0.17194 | 692 |
| Region Control: Upper East | 0.14923 | 0.09148 | 1.6313 | 1.033e-01 | -0.03038 | 0.32885 | 692 |
| Region Control: Upper West | -0.01067 | 0.08520 | -0.1253 | 9.004e-01 | -0.17794 | 0.15660 | 692 |
| Region Control: Volta | 0.22507 | 0.05901 | 3.8141 | 1.489e-04 | 0.10921 | 0.34092 | 692 |
| Region Control: Western | 0.02745 | 0.15612 | 0.1758 | 8.605e-01 | -0.27908 | 0.33399 | 692 |

*Note*. N = 708, multiple R^2^ = 0.1109, adjusted R^2^ = 0.0916, model p-value = 2.569e-14, CI = Confidence Interval, LL = Lower Limit, UL = Upper Limit, D.F = Degrees of freedom.

**Table 3 Column 3: Environmental Factors**

| **Variable** | **Estimate** | **Std. Error** | **t-value** | **P-value** | **95% CI**  **LL UL** | | **D.F** |
| --- | --- | --- | --- | --- | --- | --- | --- |
| Intercept | 0.4915623 | 0.05062 | 9.710154 | 5.473e-21 | 0.39217 | 0.5910 | 695 |
| Seen/heard something negative about vaccine | 0.0884268 | 0.03502 | 2.524824 | 1.180e-02 | 0.01966 | 0.1572 | 695 |
| Find it difficult to get vaccine | -0.1972177 | 0.03775 | -5.224452 | 2.311e-07 | -0.27133 | -0.1231 | 695 |
| Vaccinators provide enough information | 0.1778270 | 0.03616 | 4.918063 | 1.092e-06 | 0.10684 | 0.2488 | 695 |
| Region Control: Brong Ahafo | 0.0832123 | 0.06953 | 1.196762 | 2.318e-01 | -0.05330 | 0.2197 | 695 |
| Region Control: Central | 0.0617260 | 0.06743 | 0.915420 | 3.603e-01 | -0.07066 | 0.1941 | 695 |
| Region Control: Eastern | 0.0851983 | 0.06913 | 1.232389 | 2.182e-01 | -0.05054 | 0.2209 | 695 |
| Region Control: Greater Accra | 0.1456355 | 0.05223 | 2.788296 | 5.444e-03 | 0.04309 | 0.2482 | 695 |
| Region Control: Northern | -0.0002861 | 0.07479 | -0.003826 | 9.969e-01 | -0.14712 | 0.1465 | 695 |
| Region Control: Upper East | 0.1370940 | 0.08933 | 1.534662 | 1.253e-01 | -0.03830 | 0.3125 | 695 |
| Region Control: Upper West | -0.0257750 | 0.08842 | -0.291498 | 7.708e-01 | -0.19938 | 0.1478 | 695 |
| Region Control: Volta | 0.2736386 | 0.05596 | 4.890243 | 1.252e-06 | 0.16378 | 0.3835 | 695 |
| Region Control: Western | 0.0451606 | 0.13115 | 0.344350 | 7.307e-01 | -0.21233 | 0.3027 | 695 |

*Note*. N = 708, multiple R^2^ = 0.138, adjusted R^2^ = 0.1231, model p-value = 2.2e-16, CI = Confidence Interval, LL = Lower Limit, UL = Upper Limit, D.F = Degrees of freedom.

**Table 3 Column 4: Overall Model (All three categories of BDM)**

| **Variable** | **Estimate** | **Std. Error** | **t-value** | **P-value** | **95% CI**  **Lower Upper** | | **D.F** |
| --- | --- | --- | --- | --- | --- | --- | --- |
| Intercept | 0.3301960 | 0.08460 | 3.90296 | 1.044e-04 | 0.164087 | 0.49631 | 685 |
| Think polio is severe | 0.0074854 | 0.05162 | 0.14501 | 8.847e-01 | -0.093863 | 0.10883 | 685 |
| Think polio causes paralysis | 0.1292671 | 0.05585 | 2.31472 | 2.092e-02 | 0.019618 | 0.23892 | 685 |
| Think polio vaccine prevents polio | -0.0689230 | 0.05293 | -1.30220 | 1.933e-01 | -0.172844 | 0.03500 | 685 |
| Think polio vaccine is safe | 0.1120543 | 0.05334 | 2.10075 | 3.603e-02 | 0.007325 | 0.21678 | 685 |
| Traditional/religious leaders support | -0.0004324 | 0.03778 | -0.01145 | 9.909e-01 | -0.074608 | 0.07374 | 685 |
| Healthcare workers support | 0.1076626 | 0.04616 | 2.33254 | 1.996e-02 | 0.017037 | 0.19829 | 685 |
| Have HH members who do not support | -0.0499487 | 0.04817 | -1.03687 | 3.002e-01 | -0.144532 | 0.04464 | 685 |
| Need permission from HH members | -0.1053954 | 0.04746 | -2.22066 | 2.670e-02 | -0.198583 | -0.01221 | 685 |
| Trust healthcare workers | 0.0347513 | 0.03924 | 0.88563 | 3.761e-01 | -0.042292 | 0.11179 | 685 |
| [Interaction term] Have HH members who don’t support vaccine-Need permission to vaccinate | -0.0068041 | 0.07231 | -0.09409 | 9.251e-01 | -0.148788 | 0.13518 | 685 |
| Seen/heard something negative about vaccine | 0.0996339 | 0.03565 | 2.79447 | 5.344e-03 | 0.029630 | 0.16964 | 685 |
| Find it difficult to get vaccine | -0.1557458 | 0.03912 | -3.98082 | 7.601e-05 | -0.232563 | -0.07893 | 685 |
| Vaccinators provide enough information | 0.1220074 | 0.03806 | 3.20532 | 1.412e-03 | 0.047271 | 0.19674 | 685 |
| Region Control: Brong Ahafo | 0.0732523 | 0.07060 | 1.03763 | 2.998e-01 | -0.065358 | 0.21186 | 685 |
| Region Control: Central | 0.0278784 | 0.06432 | 0.43342 | 6.648e-01 | -0.098412 | 0.15417 | 685 |
| Region Control: Eastern | 0.0681820 | 0.06635 | 1.02768 | 3.045e-01 | -0.062083 | 0.19845 | 685 |
| Region Control: Greater Accra | 0.1366705 | 0.05132 | 2.66285 | 7.930e-03 | 0.035897 | 0.23744 | 685 |
| Region Control: Northern | 0.0176449 | 0.07356 | 0.23987 | 8.105e-01 | -0.126787 | 0.16208 | 685 |
| Region Control: Upper East | 0.1432078 | 0.08928 | 1.60398 | 1.092e-01 | -0.032093 | 0.31851 | 685 |
| Region Control: Upper West | -0.0361555 | 0.08253 | -0.43811 | 6.614e-01 | -0.198191 | 0.12588 | 685 |
| Region Control: Volta | 0.2002406 | 0.05594 | 3.57958 | 3.684e-04 | 0.090407 | 0.31007 | 685 |
| Region Control: Western | 0.0301813 | 0.14380 | 0.20989 | 8.338e-01 | -0.252156 | 0.31252 | 685 |

*Note*. N = 708, multiple R^2^ = 0.1917, adjusted R^2^ = 0.1658, model p-value = 2.2e-16, CI = Confidence Interval, LL = Lower Limit, UL = Upper Limit, D.F = Degrees of freedom.
