# Supplementary material for: Strengthening polio vaccine demand in Ghana: Understanding the factors influencing uptake of the vaccine and the effectiveness of different message frames
Source: PLoS One. 2023 Feb 10;18(2):e0279809. doi: 10.1371/journal.pone.0279809 (PMC9916648; doi:10.1371/journal.pone.0279809)
Supplement: S4 Table — (DOCX) [file pone.0279809.s004.docx]

**S4 Tables: Details of Regressions in Table 4**

**Table 4 Column 1: Psychological Factors**

| **Variable** | **Estimate** | **Std. Error** | **t-value** | **P-value** | **95% CI**  **LL UL** | | **D.F** |
| --- | --- | --- | --- | --- | --- | --- | --- |
| Intercept | 0.570034 | 0.09341 | 6.10232 | 4.252e-09 | 0.386005 | 0.7541 | 236 |
| Think polio is severe | 0.008259 | 0.06225 | 0.13267 | 8.946e-01 | -0.114382 | 0.1309 | 236 |
| Think polio causes paralysis | -0.018292 | 0.06741 | -0.27137 | 7.863e-01 | -0.151089 | 0.1145 | 236 |
| Think polio vaccine prevents polio | 0.046075 | 0.08039 | 0.57314 | 5.671e-01 | -0.112299 | 0.2044 | 236 |
| Think polio vaccine is safe | 0.186653 | 0.07428 | 2.51286 | 1.264e-02 | 0.040318 | 0.3330 | 236 |
| Region Control: Brong Ahafo | 0.220098 | 0.07351 | 2.99404 | 3.046e-03 | 0.075274 | 0.3649 | 236 |
| Region Control: Central | 0.092637 | 0.10140 | 0.91357 | 3.619e-01 | -0.107130 | 0.2924 | 236 |
| Region Control: Eastern | -0.105018 | 0.11445 | -0.91758 | 3.598e-01 | -0.330492 | 0.1205 | 236 |
| Region Control: Greater Accra | 0.033271 | 0.08217 | 0.40491 | 6.859e-01 | -0.128607 | 0.1951 | 236 |
| Region Control: Northern | 0.150723 | 0.08121 | 1.85593 | 6.471e-02 | -0.009269 | 0.3107 | 236 |
| Region Control: Upper East | 0.140331 | 0.10905 | 1.28688 | 1.994e-01 | -0.074501 | 0.3552 | 236 |
| Region Control: Upper West | 0.007036 | 0.11308 | 0.06222 | 9.504e-01 | -0.215739 | 0.2298 | 236 |
| Region Control: Volta | 0.018704 | 0.16059 | 0.11647 | 9.074e-01 | -0.297672 | 0.3351 | 236 |
| Region Control: Western | 0.260880 | 0.07022 | 3.71507 | 2.536e-04 | 0.122538 | 0.3992 | 236 |

*Note*. N = 250, multiple R^2^ = 0.1001, adjusted R^2^ = 0.05048, model p-value = 0.0004023, CI = Confidence Interval, LL = Lower Limit, UL = Upper Limit, D.F = Degrees of freedom.

**Table 4 Column 2: Sociological Factors**

| **Variable** | **Estimate** | **Std. Error** | **t-value** | **P-value** | **95% CI**  **LL UL** | | **D.F** |
| --- | --- | --- | --- | --- | --- | --- | --- |
| Intercept | 0.564952 | 0.09783 | 5.77500 | 2.435e-08 | 0.372218 | 0.7577 | 234 |
| Traditional/religious leaders support | 0.051251 | 0.05457 | 0.93916 | 3.486e-01 | -0.056263 | 0.1588 | 234 |
| Healthcare workers support | 0.170179 | 0.05671 | 3.00102 | 2.982e-03 | 0.058457 | 0.2819 | 234 |
| Have HH members who do not support | 0.099988 | 0.07685 | 1.30106 | 1.945e-01 | -0.051421 | 0.2514 | 234 |
| Need permission from HH members | -0.004381 | 0.07696 | -0.05693 | 9.546e-01 | -0.156004 | 0.1472 | 234 |
| Trust healthcare workers | 0.041918 | 0.05676 | 0.73850 | 4.610e-01 | -0.069910 | 0.1537 | 234 |
| [Interaction term] Have HH members who don’t support vaccine-Need permission to vaccinate | -0.106503 | 0.10792 | -0.98685 | 3.247e-01 | -0.319125 | 0.1061 | 234 |
| Region Control: Brong Ahafo | 0.200262 | 0.07658 | 2.61493 | 9.504e-03 | 0.049380 | 0.3511 | 234 |
| Region Control: Central | 0.069270 | 0.10425 | 0.66447 | 5.070e-01 | -0.136115 | 0.2747 | 234 |
| Region Control: Eastern | -0.113125 | 0.11319 | -0.99947 | 3.186e-01 | -0.336119 | 0.1099 | 234 |
| Region Control: Greater Accra | 0.042957 | 0.08198 | 0.52397 | 6.008e-01 | -0.118563 | 0.2045 | 234 |
| Region Control: Northern | 0.160458 | 0.08351 | 1.92135 | 5.590e-02 | -0.004076 | 0.3250 | 234 |
| Region Control: Upper East | 0.146047 | 0.10956 | 1.33308 | 1.838e-01 | -0.069796 | 0.3619 | 234 |
| Region Control: Upper West | 0.054692 | 0.11221 | 0.48739 | 6.264e-01 | -0.166386 | 0.2758 | 234 |
| Region Control: Volta | 0.004360 | 0.16526 | 0.02638 | 9.790e-01 | -0.321235 | 0.3300 | 234 |
| Region Control: Western | 0.254265 | 0.09099 | 2.79428 | 5.633e-03 | 0.074991 | 0.4335 | 234 |

*Note*. N = 250, multiple R^2^ = 0.1167, adjusted R^2^ = 0.06006, model p-value = 0.003661, CI = Confidence Interval, LL = Lower Limit, UL = Upper Limit, D.F = Degrees of freedom.

**Table 4 Column 3: Environmental Factors**

| **Variable** | **Estimate** | **Std. Error** | **t-value** | **P-value** | **95% CI**  **LL UL** | | **D.F** |
| --- | --- | --- | --- | --- | --- | --- | --- |
| Intercept | 0.68930 | 0.06590 | 10.4603 | 2.664e-21 | 0.55948 | 0.81912 | 237 |
| Seen/heard something negative about vaccine | -0.01029 | 0.05537 | -0.1858 | 8.528e-01 | -0.11938 | 0.09880 | 237 |
| Find it difficult to get vaccine | -0.06505 | 0.05027 | -1.2939 | 1.970e-01 | -0.16408 | 0.03399 | 237 |
| Vaccinators provide enough information | 0.19059 | 0.05236 | 3.6403 | 3.343e-04 | 0.08745 | 0.29373 | 237 |
| Region Control: Brong Ahafo | 0.22448 | 0.06971 | 3.2203 | 1.460e-03 | 0.08716 | 0.36180 | 237 |
| Region Control: Central | 0.06347 | 0.10255 | 0.6189 | 5.366e-01 | -0.13855 | 0.26550 | 237 |
| Region Control: Eastern | -0.10894 | 0.10984 | -0.9918 | 3.223e-01 | -0.32533 | 0.10744 | 237 |
| Region Control: Greater Accra | 0.01243 | 0.08003 | 0.1554 | 8.767e-01 | -0.14523 | 0.17010 | 237 |
| Region Control: Northern | 0.10411 | 0.07893 | 1.3189 | 1.885e-01 | -0.05139 | 0.25961 | 237 |
| Region Control: Upper East | 0.18235 | 0.12100 | 1.5070 | 1.331e-01 | -0.05602 | 0.42072 | 237 |
| Region Control: Upper West | 0.01197 | 0.11882 | 0.1007 | 9.199e-01 | -0.22212 | 0.24606 | 237 |
| Region Control: Volta | 0.06384 | 0.16936 | 0.3770 | 7.065e-01 | -0.26980 | 0.39748 | 237 |
| Region Control: Western | 0.31184 | 0.08753 | 3.5626 | 4.439e-04 | 0.13940 | 0.48428 | 237 |

*Note*. N = 250, multiple R^2^ = 0.1101, adjusted R^2^ = 0.06508, model p-value = 0.0002915, CI = Confidence Interval, LL = Lower Limit, UL = Upper Limit, D.F = Degrees of freedom.

**Table 4 Column 4: Overall Model (All three categories of BDM)**

| **Variable** | **Estimate** | **Std. Error** | **t-value** | **P-value** | **95% CI**  **Lower Upper** | | **D.F** |
| --- | --- | --- | --- | --- | --- | --- | --- |
| Intercept | 0.532036 | 0.11753 | 4.52676 | 9.668e-06 | 0.300444 | 0.76363 | 227 |
| Think polio is severe | -0.008226 | 0.06064 | -0.13566 | 8.922e-01 | -0.127711 | 0.11126 | 227 |
| Think polio causes paralysis | -0.024367 | 0.06749 | -0.36106 | 7.184e-01 | -0.157350 | 0.10862 | 227 |
| Think polio vaccine prevents polio | 0.037471 | 0.08206 | 0.45661 | 6.484e-01 | -0.124232 | 0.19917 | 227 |
| Think polio vaccine is safe | 0.121285 | 0.07825 | 1.55005 | 1.225e-01 | -0.032896 | 0.27547 | 227 |
| Traditional/religious leaders support | 0.012897 | 0.05439 | 0.23713 | 8.128e-01 | -0.094273 | 0.12007 | 227 |
| Healthcare workers support | 0.121818 | 0.05778 | 2.10829 | 3.610e-02 | 0.007963 | 0.23567 | 227 |
| Have HH members who do not support | 0.073825 | 0.07416 | 0.99542 | 3.206e-01 | -0.072315 | 0.21996 | 227 |
| Need permission from HH members | -0.015386 | 0.07448 | -0.20659 | 8.365e-01 | -0.162138 | 0.13137 | 227 |
| Trust healthcare workers | 0.033272 | 0.05570 | 0.59735 | 5.509e-01 | -0.076483 | 0.14303 | 227 |
| [Interaction term] Have HH members who don’t support vaccine-Need permission to vaccinate | -0.071141 | 0.10865 | -0.65476 | 5.133e-01 | -0.285238 | 0.14296 | 227 |
| Seen/heard something negative about vaccine | -0.016450 | 0.05601 | -0.29367 | 7.693e-01 | -0.126824 | 0.09392 | 227 |
| Find it difficult to get vaccine | -0.065417 | 0.05014 | -1.30460 | 1.934e-01 | -0.164223 | 0.03339 | 227 |
| Vaccinators provide enough information | 0.118829 | 0.05742 | 2.06955 | 3.963e-02 | 0.005689 | 0.23197 | 227 |
| Region Control: Brong Ahafo | 0.192469 | 0.08047 | 2.39172 | 1.758e-02 | 0.033900 | 0.35104 | 227 |
| Region Control: Central | 0.080485 | 0.10516 | 0.76534 | 4.449e-01 | -0.126736 | 0.28771 | 227 |
| Region Control: Eastern | -0.117596 | 0.10527 | -1.11711 | 2.651e-01 | -0.325024 | 0.08983 | 227 |
| Region Control: Greater Accra | 0.002744 | 0.08259 | 0.03322 | 9.735e-01 | -0.160001 | 0.16549 | 227 |
| Region Control: Northern | 0.110281 | 0.08391 | 1.31429 | 1.901e-01 | -0.055059 | 0.27562 | 227 |
| Region Control: Upper East | 0.133656 | 0.10822 | 1.23499 | 2.181e-01 | -0.079597 | 0.34691 | 227 |
| Region Control: Upper West | 0.012696 | 0.11140 | 0.11397 | 9.094e-01 | -0.206815 | 0.23221 | 227 |
| Region Control: Volta | 0.029206 | 0.16213 | 0.18014 | 8.572e-01 | -0.290264 | 0.34868 | 227 |
| Region Control: Western | 0.271519 | 0.11582 | 2.34434 | 1.992e-02 | 0.043301 | 0.49974 | 227 |

*Note*. N = 250, multiple R^2^ = 0.1665, adjusted R^2^ = 0.08568, model p-value = 0.002264, CI = Confidence Interval, LL = Lower Limit, UL = Upper Limit, D.F = Degrees of freedom.
