# Supplementary material for: Strengthening polio vaccine demand in Ghana: Understanding the factors influencing uptake of the vaccine and the effectiveness of different message frames
Source: PLoS One. 2023 Feb 10;18(2):e0279809. doi: 10.1371/journal.pone.0279809 (PMC9916648; doi:10.1371/journal.pone.0279809)
Supplement: S5 Table — (DOCX) [file pone.0279809.s005.docx]

**S5 Tables: Details of Regressions in Table 5**

**Table 5 Column 1: Effect on intention to vaccinate a child in future**

| **Variable** | **Estimate** | **Std. Error** | **t-value** | **P-value** | **95% CI** | | **D.F** |
| --- | --- | --- | --- | --- | --- | --- | --- |
|  |  |  |  |  | **LL** | **UL** |  |
| Intercept | 0.89865 | 0.02489 | 36.1026 | 2.997e-162 | 0.849778 | 0.94752 | 703 |
| Social Norms Message | 0.05484 | 0.03108 | 1.7644 | 7.810e-02 | -0.006184 | 0.11586 | 703 |
| Fear Message | 0.01744 | 0.03407 | 0.5117 | 6.090e-01 | 0.049461 | 0.08433 | 703 |
| Safety Message | 0.02588 | 0.03258 | 0.7944 | 4.272e-01 | -0.038079 | 0.08984 | 703 |
| Messenger Message | 0.03158 | 0.03357 | 0.9410 | 3.470e-01 | -0.034316 | 0.09748 | 703 |

*Note*. N = 708, multiple R^2^ = 0.004436, adjusted R^2^ = -0.001229, model p-value = 0.4687, CI = Confidence Interval, LL = Lower Limit, UL = Upper Limit, D.F = Degrees of freedom.

**Table 5 Column 2: Effect on intention to vaccinate a child in future**

| **Variable** | **Estimate** | **Std. Error** | **t-value** | **P-value** | **95% CI** | | **D.F** |
| --- | --- | --- | --- | --- | --- | --- | --- |
|  |  |  |  |  | **LL** | **UL** |  |
| Intercept | 0.74836 | 0.05035 | 14.8618 | 1.242e-43 | 0.649499 | 0.84723 | 701 |
| Social Norms Message | 0.05638 | 0.03065 | 1.8397 | 6.624e-02 | -0.003789 | 0.11655 | 701 |
| Fear Message | 0.02123 | 0.03202 | 0.6631 | 5.075e-01 | -0.041628 | 0.08409 | 701 |
| Safety Message | 0.02354 | 0.03135 | 0.7509 | 4.530e-01 | -0.038012 | 0.08510 | 701 |
| Messenger Message | 0.03309 | 0.03257 | 1.0160 | 3.100e-01 | -0.030852 | 0.09703 | 701 |
| Child has received polio vaccine | 0.02042 | 0.02075 | 0.9840 | 3.254e-01 | -0.020325 | 0.06117 | 701 |
| Will vaccinate child in next campaign | 0.15922 | 0.04107 | 3.8765 | 1.159e-04 | 0.078577 | 0.23986 | 701 |

*Note*. N = 708, multiple R^2^ = 0.05231, adjusted R^2^ = 0.0442, model p-value = 0.009475, CI = Confidence Interval, LL = Lower Limit, UL = Upper Limit, D.F = Degrees of freedom.

**Table 5 Column 3: Effect on willingness to recommend vaccination to others**

| **Variable** | **Estimate** | **Std. Error** | **t-value** | **P-value** | **95% CI** | | **D.F** |
| --- | --- | --- | --- | --- | --- | --- | --- |
|  |  |  |  |  | **LL** | **UL** |  |
| Intercept | 0.945946 | 0.01865 | 50.7198 | 4.124e-237 | 0.90933 | 0.98256 | 703 |
| Social Norms Message | -0.023465 | 0.03011 | -0.7794 | 4.360e-01 | -0.08258 | 0.03565 | 703 |
| Fear Message | 0.005103 | 0.02599 | 0.1963 | 8.444e-01 | -0.04593 | 0.05614 | 703 |
| Safety Message | -0.021418 | 0.02810 | -0.7623 | 4.462e-01 | -0.07658 | 0.03375 | 703 |
| Messenger Message | -0.007961 | 0.02832 | -0.2811 | 7.787e-01 | -0.06357 | 0.04765 | 703 |

*Note*. N = 708, multiple R^2^ = 0.002181, adjusted R^2^ = -0.003497, model p-value = 0.8192, CI = Confidence Interval, LL = Lower Limit, UL = Upper Limit, D.F = Degrees of freedom.

**Table 5 Column 4: Effect on willingness to recommend vaccination to others**

| **Variable** | **Estimate** | **Std. Error** | **t-value** | **P-value** | **95% CI** | | **D.F** |
| --- | --- | --- | --- | --- | --- | --- | --- |
|  |  |  |  |  | **LL** | **UL** |  |
| Intercept | 0.789366 | 0.04729 | 16.6903 | 6.567e-53 | 0.696509 | 0.88222 | 701 |
| Social Norms Message | -0.021171 | 0.02929 | -0.7229 | 4.700e-01 | -0.078670 | 0.03633 | 701 |
| Fear Message | 0.008185 | 0.02484 | 0.3296 | 7.418e-01 | -0.040575 | 0.05694 | 701 |
| Safety Message | -0.020720 | 0.02631 | -0.7875 | 4.313e-01 | -0.072380 | 0.03094 | 701 |
| Messenger Message | -0.007322 | 0.02632 | -0.2782 | 7.809e-01 | -0.058989 | 0.04435 | 701 |
| Child has received polio vaccine | 0.045099 | 0.02075 | 2.1737 | 3.006e-02 | 0.004364 | 0.08583 | 701 |
| Will vaccinate child in next campaign | 0.147315 | 0.04056 | 3.6317 | 3.021e-04 | 0.067675 | 0.2269 | 701 |

*Note*. N = 708, multiple R^2^ = 0.06028, adjusted R^2^ = 0.05224, model p-value = 0.0007287, CI = Confidence Interval, LL = Lower Limit, UL = Upper Limit, D.F = Degrees of freedom.
