# Supplementary material for: Strengthening polio vaccine demand in Ghana: Understanding the factors influencing uptake of the vaccine and the effectiveness of different message frames
Source: PLoS One. 2023 Feb 10;18(2):e0279809. doi: 10.1371/journal.pone.0279809 (PMC9916648; doi:10.1371/journal.pone.0279809)
Supplement: S6 Table — (DOCX) [file pone.0279809.s006.docx]

**S6 Tables: Details of Regressions in Table 6**

**Table 6 Column 1: Intention to vaccinate a child**

| **Variable** | **Estimate** | **Std. Error** | **t-value** | **P-value** | **95% CI** | | **D.F** |
| --- | --- | --- | --- | --- | --- | --- | --- |
|  |  |  |  |  | **LL** | **UL** |  |
| Intercept | 0.4545 | 0.1575 | 2.8868 | 0.005819 | 0.13795 | 0.7711 | 48 |
| Social Norms Message | 0.4545 | 0.1818 | 2.5000 | 0.015890 | 0.08898 | 0.8201 | 48 |
| Fear Message | 0.1288 | 0.2165 | 0.5948 | 0.554801 | -0.30659 | 0.5642 | 48 |
| Safety Message | 0.4545 | 0.1818 | 2.5000 | 0.015890 | 0.08898 | 0.8201 | 48 |
| Messenger Message | 0.4205 | 0.2010 | 2.0914 | 0.041812 | 0.01623 | 0.8247 | 48 |

*Note*. N = 53, multiple R^2^ = 0.1907, adjusted R^2^ = 0.1233, model p-value = 0.05407, CI = Confidence Interval, LL = Lower Limit, UL = Upper Limit, D.F = Degrees of freedom.

**Table 6 Column 2: Willingness to recommend vaccination to others**

| **Variable** | **Estimate** | **Std. Error** | **t-value** | **P-value** | **95% CI** | | **D.F** |
| --- | --- | --- | --- | --- | --- | --- | --- |
|  |  |  |  |  | **LL** | **UL** |  |
| Intercept | 0.5455 | 0.1575 | 3.4641 | 0.00113 | 0.22886 | 0.8620 | 48 |
| Social Norms Message | 0.2727 | 0.1992 | 1.3693 | 0.17728 | -0.12773 | 0.6732 | 48 |
| Fear Message | 0.3712 | 0.1782 | 2.0837 | 0.04254 | 0.01302 | 0.7294 | 48 |
| Safety Message | 0.2727 | 0.1992 | 1.3693 | 0.17728 | -0.12773 | 0.6732 | 48 |
| Messenger Message | 0.2045 | 0.2271 | 0.9006 | 0.37227 | -0.25209 | 0.6612 | 48 |

*Note*. N = 53, multiple R^2^ = 0.09333, adjusted R^2^ = 0.01777, model p-value = 0.3481, CI = Confidence Interval, LL = Lower Limit, UL = Upper Limit, D.F = Degrees of freedom.
